# Circulating DNA methylation level of CXCR5 correlates with inflammation in patients with rheumatoid arthritis

**DOI:** 10.1002/iid3.902

**Published:** 2023-06-23

**Authors:** Yiming Shi, Cen Chang, Lingxia Xu, Ping Jiang, Kai Wei, Jianan Zhao, Linshuai Xu, Yehua Jin, Runrun Zhang, Huijuan Wang, Yi Qian, Yingying Qin, Qin Ding, Ting Jiang, Shicheng Guo, Rongsheng Wang, Dongyi He

**Affiliations:** ^1^ Department of Rheumatology, Shanghai Guanghua Hospital Shanghai University of Traditional Chinese Medicine Shanghai China; ^2^ Guanghua Clinical Medical College Shanghai University of Traditional Chinese Medicine Shanghai China; ^3^ Institute of Arthritis Research in Integrative Medicine Shanghai Academy of Traditional Chinese Medicine Shanghai China; ^4^ Department of Rheumatology The Second Affiliated Hospital of Zhejiang Chinese Medical University Hangzhou China; ^5^ Computation and Informatics in Biology and Medicine University of Wisconsin‐Madison Madison Wisconsin USA; ^6^ Department of Medical Genetics, School of Medicine and Public Health University of Wisconsin‐ Madison Madison Wisconsin USA

**Keywords:** cg04537602, CXCR5, DNA methylation, rheumatoid arthritis

## Abstract

**Objectives:**

To assess the differences in circulating DNA methylation levels of CXCR5 between rheumatoid arthritis (RA) and osteoarthritis (OA) and healthy controls (HC), and the correlation of methylation changes with clinical characteristics of RA patients.

**Methods:**

Peripheral blood samples were collected from 239 RA patients, 30 patients with OA, and 29 HC. Target region methylation sequencing to the promoter region of CXCR5 was achieved using MethylTarget. The methylation level of cg04537602 and methylation haplotype were compared among the three groups, and the correlation between methylation levels and clinical characteristics of RA patients was performed by Spearman's rank correlation analysis.

**Results:**

The methylation level of cg04537602 was significantly higher in the peripheral blood of RA patients compared with OA patients (*p* = 1.3 × 10^−3^) and in the HC group (*p* = 5.5 × 10^−^^4^). The sensitivity was enhanced when CXCR5 methylation level combined with rheumatoid factor and anti–cyclic citrullinated peptide with area under curve (AUC) of 0.982 (95% confidence interval 0.970–0.995). The methylation level of cg04537602 in RA was positively correlated with C‐reactive protein (CRP) (*r* = .16, *p* = .01), and in RA patients aged 60 years and above, cg04537602 methylation levels were positively correlated with CRP (*r* = .31, *p* = 4.7 × 10^−^^4^), tender joint count (*r* = .21, *p* = .02), visual analog scales score (*r* = .21, *p* = .02), Disease Activity Score in 28 joints (DAS28) using the CRP level DAS28‐CRP (r = .27, *p* = 2.1 × 10^−^^3^), and DAS28‐ESR (*r* = .22, *p* = .01). We also observed significant differences of DNA methylation haplotypes in RA patients compared with OA patients and HC, which was consistent with single‐loci‐based CpG methylation measurement.

**Conclusion:**

The methylation level of CXCR5 was significantly higher in RA patients than in OA and HC, and correlated with the level of inflammation in RA patients, our study establishes a link between CXCR5 DNA methylation and clinical features that may help in the diagnosis and disease management of RA patients.

## Graphical abstract

The methylation level of CXCR5 was significantly higher in rheumatoid arthritis(RA) patients than in osteoarthritisand HC, and correlated with the level of inflammation in RA patients, Our study establishes a link between CXCR5 DNA methylation and clinical features that may help in the diagnosis and disease management of RA patients.

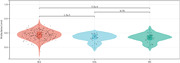

## 1 INTRODUCTION

Rheumatoid arthritis (RA) is a complex chronic systemic inflammatory disease, one of the most common autoimmune diseases that affects mainly the joints, causing cartilage and bone damage, leading to disability, and is associated with a range of extra‐articular manifestations and comorbidities in addition to joint involvement.^1^, ^2^ The exact cause of RA is not fully understood, but recent evidence suggests that epigenetics plays an important role in the development of RA.^3^, ^4^, ^5^ DNA methylation is the most common study of epigenetic modification, and methylation variation analysis has been used to characterize different aspects of RA pathology.^6^, ^7^

Chemokines and their receptors play an important role in cell migration and inflammation in chronic inflammatory diseases.^8^ CXCR5 is highly expressed in mature circulating B lymphocytes, follicle helper T cells (TfH), and skin‐derived migrating dendritic cells (DC) and controls their migration to CXCL13, and is the surface markers of TfH cells.^9^ CXCL13 is the only ligand for CXCR5 and the CXCL13/CXCR5 axis is associated with the development of tumors, autoimmune diseases, infectious diseases, and many other diseases.^10^, ^11^ CXCR5 is upregulated in RA synovial tissue and peripheral blood,^12^, ^13^ involved in germinal center (GC) formation and attracting endothelial progenitor cells (EPCs) for homing and angiogenesis.^14^ CXCR5 polymorphisms are associated with RA sensitivity.^15^ Immune abnormalities of T and B lymphocytes are important in the pathogenesis of RA. Recent studies have demonstrated an increased frequency of circulating CXCR5^+^ T cells in the peripheral blood of RA patients, which correlates with serum anticitrullinated‐protein antibody (ACPA) levels and disease severity.^16^ In addition, lymphoid tissue biopsies from RA patients showed that the frequencies of CD4^+^ CXCR5^+^ follicular T cells and CD8^+^ CXCR5^+^ follicular T cells were also significantly increased in patients with early RA.^17^ B10 cells can secrete interleukin‐10 (IL‐10) to exert an anti‐inflammatory effect.^18^ However, recent studies have shown that in RA patients, CXCR5 levels on the surface of B cells are reduced and that the migration of RA B10 cells into CXCL13‐rich synovial fluid (SF) is reduced, promoting an inflammatory response in the joint.^19^ CXCR5‐mediated colocalization of Tfh and B cells in secondary lymphoid organs (SLOs) is absolutely essential for RA induction.^20^, ^21^ Therapies targeting Tfh cells are also gaining attention in immunoglobulin 4‐related diseases.^22^ Another study found that chimeric antigen receptor‐T (CAR‐T) cells targeting CXCR5 can remove tumor cells with high CXCR5 expression from B cell follicles of lymphoid organs, especially in the treatment of follicular lymphoma and chronic lymphocytic leukemia.^23^ CXCR5 and Tfh cells may be promising therapeutic targets for the treatment of RA.^21^, ^24^

As mentioned above, CXCR5 plays an important role in the pathology of RA. However, the role of methylation levels of the CXCR5 in RA has not been well investigated. In this study, we analyzed circulating DNA methylation levels of CXCR5 in patients with RA, OA, and HC. We depicted the level of CXCR5 DNA methylation and the proportion of haplotypes in the peripheral blood of RA patients, combined the methylation changes with clinical characteristics of RA patients for correlation analysis, explored the role of CXCR5 in RA from an epigenetic perspective, and helped to deepen the understanding of the pathogenesis of RA.

## 2 MATERIALS AND METHODS

### 2.1 Study design and patients

The study included 239 RA patients, 30 patients with OA, and 29 healthy controls (HC), all from Precision Medicine Research Cohort in Shanghai Guanghua Hospital.^25^ All RA patients satisfied the American College of Rheumatology (ACR)/European League Against Rheumatism (EULAR) classification criteria 2010.^26^ All OA patients met the ACR clinical classification criteria for osteoarthritis.^27^ Patients younger than 18 years of age, with other rheumatic diseases or the presence of acute or chronic infections or tumors and those who are preparing for or during pregnancy were excluded. HC is selected from the Shanghai Guanghua Hospital of Integrated Chinese and Western Medicine after passing a health checkup. Peripheral blood samples were collected from participants and stored at −80°C, and basic information on each participant and clinical information of RA patients were collected.

### 2.2 Methylome profiling

Peripheral venous blood was collected from all participants and genomic DNA was extracted using the Blood Genomic DNA Extraction Kit (Concert, RC1001), a 1.5 mL sample tube was pretreated with 40 μL of Proteinase K solution at a concentration of 10 mg/mL, followed by 400 μL of the whole blood sample. The pretreated samples were subjected to automated DNA extraction in a nucleic acid purifier (Concert, H8/HF16) (Supporting Information 2). Sample quality control required concentrations ≥20 ng/μL and sample purity ≥400 ng in total (OD260/280 = 1.7–1.9, OD260/230 ≥ 2.0). DNA was treated with bisulfite. Targeted methylation profiling is applied for methylation measurement for a specific 200–500 bp region.^28^, ^29^ Methylation Primer software was used for the primer design of the methylation loci region (Primer F:AGAGATAGTTAGGAATAGGAAGATTTATGAG, Primer R:ACCTACTTAATACAAAAACCCATCTATTC). Two polymerase chain reaction (PCR) amplifications were performed, the first one was a PCR amplification with specific primers for the target region of the bisulfite transformed sample, the sample target region was enriched using the optimized system. The second time was Index PCR, which aims to mix all the target region enrichment products of the sample and then amplify them, and using primers with Index sequences to introduce specific tag sequences compatible with the Illumina HiSeq (Illumina) platform to the end of the library (Supporting Information 2). PCR products were separated by agarose electrolysis and purified, followed by high‐throughput sequencing on the Illumina HiSeq platform using 2 × 150 bp double‐end sequencing mode to obtain FastQ data.

### 2.3 Statistical analysis

IBM SPSS Statistics (Version 27; IBM Corp.) and Sangerbox^30^ was used for data analysis. Kruskal–Wallis rank sum test was used to compare the differences between variables in different groups; Spearman's rank correlation analysis was used to evaluate the relationship between DNA methylation levels and clinical data of RA patients. *p* < .05 were regarded as statistically significant.

## 3 RESULTS

### 3.1 Demographic and clinical characteristics of participants

239 participants with RA, 30 with OA, and 29 with HC were included in the analysis. Basic characteristics are listed in Table 1. All participants were female and there were no significant differences in height, weight, or age between the three groups (*p* > .05).

**Table 1:** The basic characteristics of participants.

Items/group	RA	OA	HC	*p* Value
Female, *n*	239	30	29	‐
Height (cm), median (IQR)	160 (156–163)	160 (154.75–162)	160 (157.5–164)	.405
Weight (kg), median (IQR)	59 (53–64)	61 (55–65)	60 (54.5–67.5)	.445
Age (years), median (IQR)	60 (52–67)	61.5 (55.75–65.25)	61 (51–64)	.44
Age ≥60, *n* (%)	124 (51.88%)	20 (66.67%)	17 (58.62%)	‐
Age <60, *n* (%)	115 (48.12%)	10 (33.33%)	12 (41.38%)	‐
RF positive, *n* (%)	204 (85.36%)	‐	‐	‐
CCP positive, *n* (%)	217 (90.79%)	‐	‐	‐
RF^+^CCP^+^, *n* (%)	193 (80.75%)	‐	‐	‐
RF^−^CCP^−^, *n* (%)	11 (4.60%)	‐	‐	‐
ESR (mm/h), median (IQR)	19 (10–30)	10 (6.5–15.75)	‐	‐
CRP (mg/L), median (IQR)	2.24 (0.65–8.51)	0.5 (0.5–0.89)	‐	‐
TJC, median (IQR)	2 (0–4)	‐	‐	‐
SJC, median (IQR)	0 (0–2)	‐	‐	‐
VAS, median (IQR)	3 (2–5)	‐	‐	‐
DAS28‐ESR, median (IQR)	3.2 (2.3–4.2)	‐	‐	‐
DAS28‐CRP, median (IQR)	2.7 (1.8–3.5)	‐	‐	‐

Abbreviations: CCP, anti–cyclic citrullinated peptide; CRP, C‐reactive protein; DAS28‐CRP, 28‐joint Disease Activity Score–C‐reactive protein; DAS28‐ESR, 28‐joint Disease Activity Score–erythrocyte sedimentation rate; ESR, erythrocyte sedimentation rate; IQR, interquartile range; RF, rheumatoid factor; SJC, swollen joint count; TJC, tender joint count; VAS score, visual analog scales score.

### 3.2 Increased CXCR5 methylation in RA patients compared with OA patients and HC

**Figure 1:**
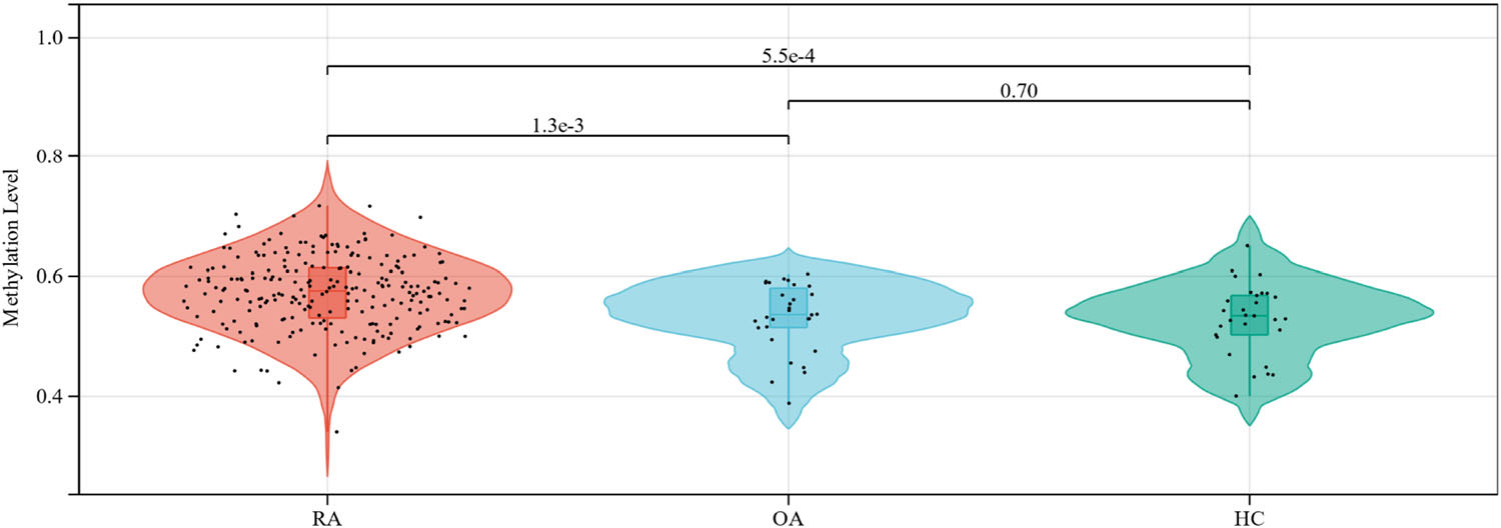
Methylation level of cg04537602 between RA patients and OA patients and HC. HC, healthycontrols; OA, osteoarthritis; RA, rheumatoid arthritis.

**Figure 2:**
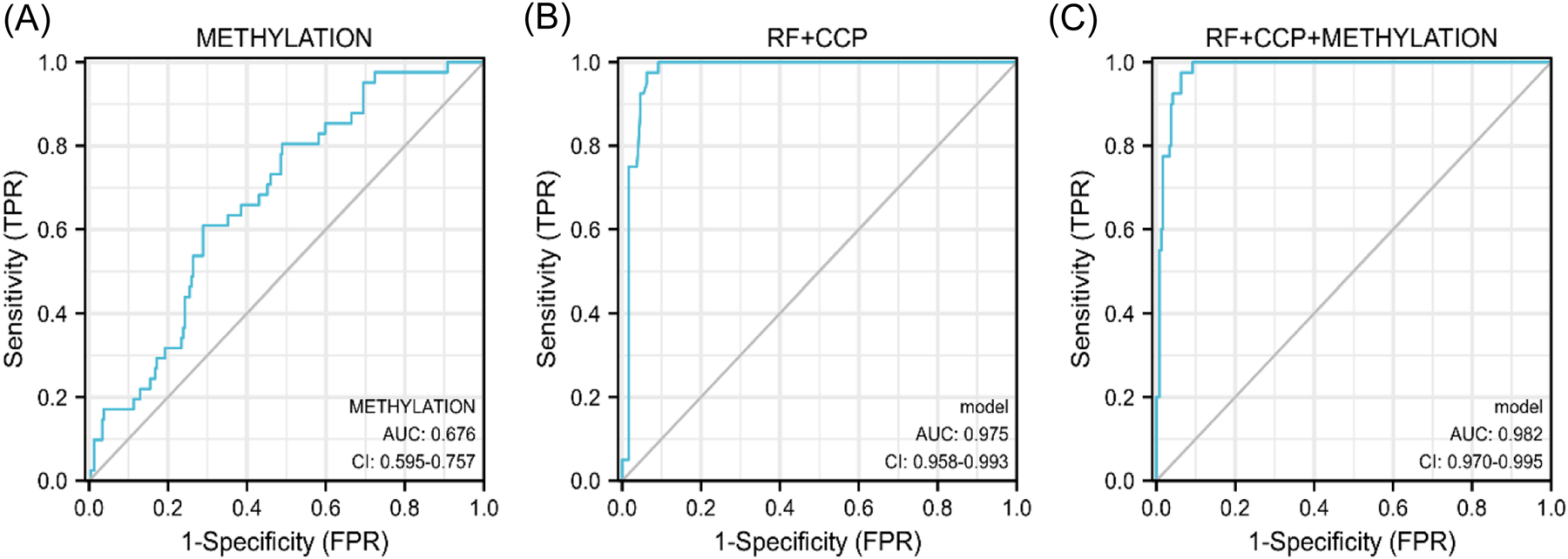
The ROC curve plot. (A) Diagnostic effectiveness of CXCR5 methylation level; (B) diagnostic effectiveness of RF and CCP; (C) diagnostic effectiveness of CXCR5 methylation levels combined with RF and CCP. CCP, anti–cyclic citrullinated peptide; RF, rheumatoid factor; ROC, receiver operating characteristic curve.

Methylation levels of CXCR5 cg04537602 in peripheral blood of RA patients were found to be significantly increased compared to patients with OA (*p* = 1.3 × 10^−3^) and HC (*p* = 5.5 × 10^−4^) (Figure 1a). Our data showed that the area under curve (AUC) of CXCR5 methylation level alone was 0.67 (95% CI 0.595–0.757) (Figure 2a). The AUC of rheumatoid factor (RF) combined anti–cyclic citrullinated peptide (CCP) was 0.975 (95% CI 0.958–0.993). The sensitivity was enhanced when CXCR5 methylation level combined with RF and CCP with AUC of 0.982 (95% CI 0.970–0.995) (Figure 2b,c).

### 3.3 Correlation analysis between the methylation level of cg04537602 in peripheral blood and clinical characteristics in patients with RA

**Figure 3:**
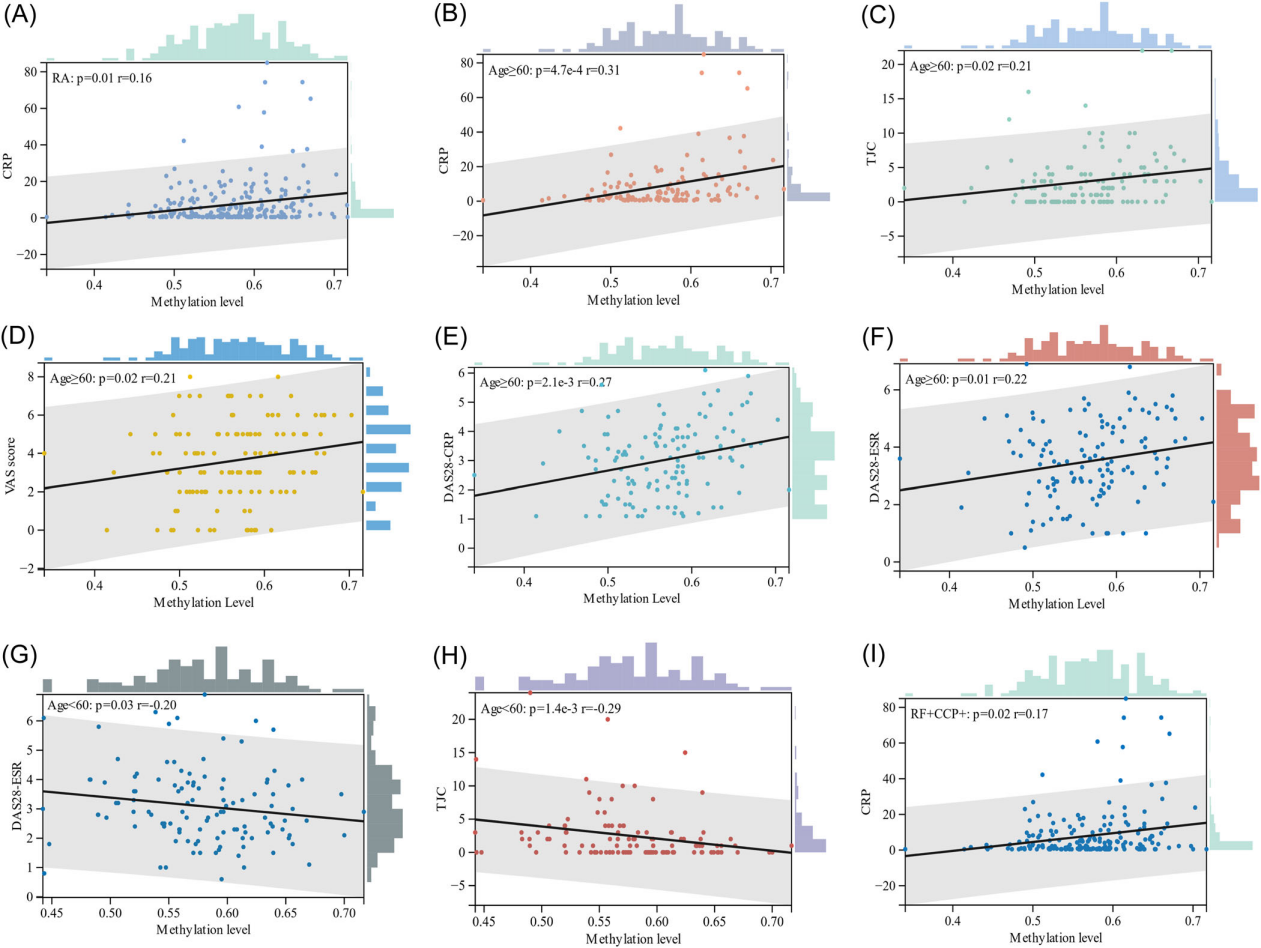
Correlation analysis between the average methylation levels of cg04537602 and clinical indicators of RA patients. (A) Correlation between methylation level and CRP; (B–F) the plot of age ≥60 group; (G, H) the plot of age <60 group; (I) the plot of RF^+^CCP^+^ group. CRP, C‐reactive protein; DAS28‐CRP, 28‐joint Disease Activity Score–C‐reactive protein; DAS28‐ESR, 28‐joint Disease Activity Score–erythrocyte sedimentation rate; TJC, tender joint count; VAS score, visual analog scales score.

The average cg04537602 methylation levels showed a positive correlation with CRP in RA group (*r* = .16, *p* = .01) (Figure 3a and Supporting Information: Table 1). RA patients were then stratified to explore the correlation of methylation levels with clinical parameters in different subgroups of RA. In RA patients aged 60 years and above, cg04537602 methylation levels were positively correlated with CRP (*r* = .31, *p* = 4.7 × 10^−4^), tender joint count (TJC) (*r* = .21, *p* = .02), visual analog scales score (*r* = .21, *p* = .02), DAS28‐CRP (*r* = .27, *p* = 2.1 × 10^−^^3^), and DAS28‐ESR (*r* = .22, *p* = .01). In RA patients aged less than 60 years, cg04537602 methylation levels were negatively correlated with DAS28‐ESR (*r* = −.20, *p* = .03), TJC (*r* = −.29, *p* = 1.4 × 10^−^^3^) (Figure 3b–h). Classification of RA patients according to RF and CCP levels, the methylation level of cg04537602 was positively correlated with CRP in RA patients with both positive RF and CCP (*r* = .17, *p* = .02) (Figure 3i).

### 3.4 Differences of methylation levels at different sites in cg04537602 between RA patients, OA patients, and HC

**Figure 4:**
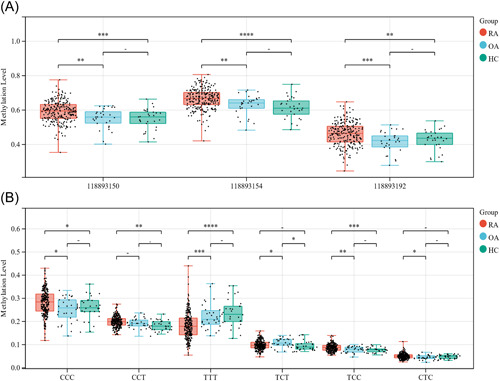
Methylation levels at different sites in cg04537602 and haplotype methylation analysis. (A) Methylation levels at differential sites between RA and OA, RA and HC, OA and HC; (B) proportion of haplotypes between and within the RA, OA, HC groups (*p＜.05; **p＜.01; ***p＜.001; ^‐^ *p*＞.05). HC, healthy controls; OA, osteoarthritis; RA, rheumatoid arthritis.

Targeted methylation sequencing can measure multiple CpG loci within ~300 bp regions. In this study, three methylation sites: 118893150, 118893154, and 118893192 are included nearby our candidate CpG site: cg04537602. Comparing the methylation levels of the three sites, significant differences were found between RA and OA, RA and HC, but not between OA and HC. The methylation levels of the three sites of RA patients were significantly higher than those of OA patients and HC (*p* < .01) (Figure 4a).

### 3.5 Haplotype methylation analysis

We found six methylation haplotypes from our methylation sequencing data including CCC, CCT, TTT, TCT, TCC, and CTC (Supporting Information: Figure 1). Analysis of the differences between the three groups for each haplotype. Compared with the OA group or HC group, the proportion of haplotypes CCC (*p* < .05) CTC (*p* < .05) and TCC (*p* < .01) increased significantly. Compared with OA group or HC group, the proportion of haplotypes TTT decreased significantly (*p* < .001). Compared with OA group, the proportion of haplotypes TCT decreased significantly (*p* < .05) (Figure 4b). Correlation analysis of haplotypes with clinical indicators showed a weak negative correlation between TTT and CRP (*r* = −.17, *p* =  6.8 × 10^−^^3^) and a weak positive correlation between CCT and CRP (*r* = .19, *p* = 3.4 × 10^−^^9^) (Supporting Information: Table 2).

## 4 DISCUSSION

In this study, we measured altered DNA methylation of CXCR5 in the peripheral blood of RA patients, OA patients, and HC. In our results, overall methylation levels were significantly higher in RA patients than in OA and HC. This result is in some agreement with that reported in another paper, which reports that CXCR5 cg04537602 locus methylation levels differ significantly between healthy individuals and patients with undifferentiated arthritis, and that a large proportion of patients with undifferentiated arthritis progress to RA, suggesting that CXCR5 methylation may be involved in the pathogenesis of RA.^31^ Methylation is an important epigenetic modification that regulates gene expression.^32^ CXCR5 is located on chromosome 11, and the regions we examined were promoter regions. Unfortunately, no CpG island was found in this region and it is unclear whether methylation at this site affects gene expression.

Circulating levels of CXCR5 methylation in RA patients were further analyzed to determine the presence of methylation variants associated with the clinical features of RA. Age is an important risk factor for many chronic inflammatory diseases, including RA and has an impact on patient disease status, dosing regimens, and so on. Moreover, in RA, a growing trend is to stratify patients according to age.^33^, ^34^ Both intrinsic and adaptive immunity change with age, with immune senescence progressing rapidly in RA patients.^34^, ^35^ Analysis of age‐related changes in lymphocytes at the molecular level revealed that CXCR5 was highly expressed in CD4 T cells of aged mice compared to young mice.^37^ Our study stratified RA patients by age above or below 60 years and found higher levels of CXCR5 circulating methylation and higher levels of CRP, TJC, VAS Score, DAS28‐ESR, and DAS28‐CRP in RA patients older than 60 years, which typically represent the patient's level of inflammation and disease activity.^38^, ^39^ Elevated physiological ESR in disease subgroups of elderly patients with RA may affect the evaluation of disease activity (mainly DAS28‐ESR) increasing the complexity of disease management.^40^ CXCR5 methylation levels may provide a new disease evaluation reference for elderly RA patients. In addition, we also tried to stratify patients according to the degree of disease activity and age of onset, but unfortunately did not find a significant correlation with methylation levels. In RA, RF and CCP antibodies are the most common, which are pathological products and have high diagnostic values.^41^ Tfh cells can assist B cells in producing high‐affinity antibodies. However, no direct correlation between CXCR5 methylation levels and antibody levels was found in our results, but in patients both positive for RF and CCP, higher levels of cg04536702 methylation were associated with higher levels of CRP. To our knowledge, our study is the first to characterize the DNA methylation levels and clinical features of CXCR5 in peripheral blood. However, the degree of correlation presented in our results is weaker, considering that DNA methylation is influenced by cell type heterogeneity, future studies may delve into the DNA methylation level of individual cells to investigate.

In the 300 bp region near cg04537602, we detected a total of three CpG loci, including 11893150, 11893154, and 11893192, and five haplotypes, including CCC, CCT, TTT, TCT, TCC, and CTC. We found that the methylation levels at all three cpg loci were significantly higher in RA patients than in OA patients and HC, and the proportion of TTT representing unmethylated status was significantly lower in RA and the proportion of CCC representing all methylated status was significantly higher, which was basically consistent with the analysis of individual CpGs. Correlation analysis of haplotypes with clinical characteristics of RA patients revealed a significant negative correlation between TTT and CPR, and a significant positive correlation between CCT and CRP. DNA methylation haplotype analysis provides a new vision for observing the direction of methylation change.^42^ The changes in methylation haplotypes in our results may suggest inflammation‐related alterations in RA.

There are limitations to our study, as confounding factors such as drug selection and different stages of the disease may have an impact on methylation levels, and our results need to be validated in a larger cohort. In conclusion, our study established a link between circulating DNA methylation levels of CXCR5 and inflammation in patient with RA and may contribute to the clinical integration of DNA methylation profiles for better classification and management of RA patients.

## AUTHOR CONTRIBUTIONS

**Dongyi He**: Conceptualization (lead); data curation (equal); funding acquisition (lead); project administration (lead); resources (lead); supervision (lead); writing—review and editing (equal). **Rongsheng Wang**: Conceptualization (equal); data curation (supporting); supervision (equal); writing—review and editing (equal). **Shicheng Guo**: Conceptualization (equal); data curation (equal); supervision (equal); writing—review and editing (lead). Yiming shi: data curation (lead); formal analysis (equal); investigation (equal); methodology (equal); visualization (lead); writing—original draft (lead). **Cen Chang**: Data curation (equal); formal analysis (equal); investigation (supporting); methodology (supporting); visualization (supporting); writing—original draft (equal). **Lingxia Xu and Ping Jiang**: Formal analysis (equal); investigation (equal); methodology (supporting). **Kai Wei, Jianan Zhao, Linshuai Xu, Yehua Jin, Runrun Zhang**, and **Huijuan Wang**: Investigation (supporting). **Yi Qian, Yingying Qin, Qin Ding**, and **Ting Jiang**: Writing—review and editing (supporting). All authors agreed to the published version of the manuscript.

## CONFLICT OF INTEREST STATEMENT

The authors declare no conflict of interest.

## ETHICS STATEMENT

This study was approved by the Ethics Committee of Guanghua Hospital of Integrated Traditional Chinese and Western Medicine (2020‐K‐06‐01) and written consent was collected before the surgery from the patients.

## Supporting information

Supporting information.

Supporting information.

## Data Availability

Data available on request from the authors.
