# Supplementary material for: Circulating DNA methylation level of CXCR5 correlates with inflammation in patients with rheumatoid arthritis
Source: Immun Inflamm Dis. 2023 Jun 23;11(6):e902. doi: 10.1002/iid3.902 (PMC10288483; doi:10.1002/iid3.902)
Supplement: Supplementary file 1 — Supporting information. [file IID3-11-e902-s002.doc]

**Supplementary material 1**

**Figure S1 The six haplotypes of cg04537602.**

**
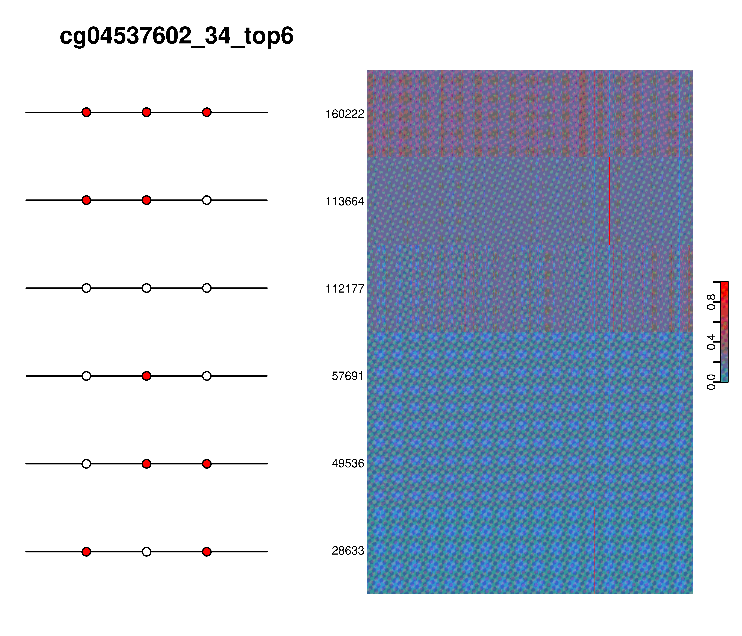
**

Hollow circles indicate t (unmethylated modification) and red circles indicate c (methylated modification).The number after each haplotype indicates the total number of reads supporting that haplotype in all samples. The heat map on the right shows the percentage of the number of reads corresponding to each haplotype.

**Table S1 Correlation analysis of the methylation level with clinical indicators in RA patients**

|  | Methylation level of cg04537602 |
| --- | --- |
| Height | 0.19(-0.09) |
| Weight | 0.50(-0.04) |
| Age | 0.43(-0.05) |
| Course | 0.15(-0.09) |
| Age of onset | 0.52(0.04) |
| TJC | 0.70(-0.02) |
| SJC | 0.93(5.6×10^-3) |
| VAS score | 0.50(0.04) |
| ESR | 0.16(0.09) |
| CRP | **0.01(0.16)** |
| RF | 0.86(-0.01) |
| CCP | 0.56(-0.04) |
| DAS28-ESR | 0.76(0.02) |
| DAS28-CRP | 0.46(0.05) |

TJC=Tender joint count,SJC=Swollen joint count,VAS score= Visual analogue scales score, ESR=erythrocyte sedimentation rate,CRP=C-reactive protein,RF=rheumatoid factor,CCP= cyclic citrullinated peptide,DAS28-ESR=28-joint Disease Activity Score-erythrocyte sedimentation rate,DAS28-CRP=28-joint Disease Activity Score- C-reactive protein. The meaning of numbers is P value( correlation coefficient).

**Table S2 Correlation analysis of haplotypes with clinical indicators**

|  | CCC | CCT | TTT | TCT | TCC | CTC |
| --- | --- | --- | --- | --- | --- | --- |
| Height | 0.22(-0.08) | 0.27(-0.07) | 0.31(0.07) | 0.67(0.03) | 0.73(-0.02) | 0.94(-5.0×10^-3) |
| Weight | 0.61(-0.03) | 0.62(-0.03) | 0.41(0.05) | 0.82(-0.01) | 0.30(-0.07) | 0.46(-0.05) |
| Age | 0.19(-0.08) | 0.05(0.12) | 0.97(2.5×10^-3) | 0.18(0.09) | 0.38(-0.06) | 0.07(-0.12) |
| Course | **0.04(-0.13)** | 0.30(0.07) | 0.63(0.03) | **0.02(0.15)** | 0.32(-0.06) | 0.29(-0.07) |
| Age of onset | 0.47(0.05) | 0.76(0.02) | 0.64(-0.03) | 0.40(-0.05) | 0.63(0.03) | 0.54(-0.04) |
| TJC | 0.83(-0.01) | 0.93(5.5×10^-3) | 0.79(0.02) | 0.69(0.03) | 0.83(0.01) | 0.10(-0.11) |
| SJC | 0.63(-0.03) | 0.47(0.05) | 0.45(-0.05) | 0.37(0.06) | 0.50(0.04) | 0.62(0.03) |
| VAS score | 0.38(0.06) | 0.84(0.01) | 0.44(-0.05) | 0.87(0.01) | 0.54(0.04) | 0.46(-0.05) |
| ESR | 0.48(0.05) | 0.35(0.06) | 0.06(-0.12) | 0.62(0.03) | 0.25(0.07) | 0.60(0.03) |
| CRP | 0.10(0.11) | **3.4e×10^-9 (0.19)** | **6.8×10^-3 (-0.17)** | 0.58(-0.04) | 0.12(0.10) | 0.79(0.02) |
| RF | 0.39(-0.06) | 0.04(0.13) | 0.77(-0.02) | 0.48(0.05) | 0.64(-0.03) | 0.75(-0.02) |
| CCP | 0.40(-0.05) | 0.82(-0.01) | 0.95(4.0×10^-3) | 0.19(0.09) | 0.93(5.5×10^-3) | 0.25(-0.08) |
| DAS28-ESR | 0.98(1.9×10^-3) | 0.67(0.03) | 0.42(-0.05) | 0.39(0.06) | 0.31(0.07) | 0.55(-0.04) |
| DAS28-CRP | 0.56(0.04) | 0.39(0.06) | 0.30(-0.07) | 0.58(-0.04) | 0.40(0.06) | 0.58(-0.04) |

TJC=Tender joint count,SJC=Swollen joint count,VAS score=Visual analogue scales score, ESR=erythrocyte sedimentation rate,CRP=C-reactive protein,RF=rheumatoid factor,CCP= cyclic citrullinated peptide,DAS28-ESR=28-joint Disease Activity Score-erythrocyte sedimentation rate,DAS28-CRP=28-joint Disease Activity Score-C-reactive protein. The meaning of numbers is P value( correlation coefficient).
