# Supplementary material for: Circulating DNA methylation level of CXCR5 correlates with inflammation in patients with rheumatoid arthritis
Source: Immun Inflamm Dis. 2023 Jun 23;11(6):e902. doi: 10.1002/iid3.902 (PMC10288483; doi:10.1002/iid3.902)
Supplement: Supplementary file 2 — Supporting information. [file IID3-11-e902-s001.doc]

**Supplementary material 2**

**Blood genomic DNA extraction steps**

1. Sample pretreatment:

Using the Blood Genomic DNA Extraction Kit (Concert, RC1001), a 1.5 mL sample tube was pretreated with 40 μL of Proteinase K solution at a concentration of 10 mg/mL, followed by 400 μL of whole blood sample.

2. The pretreated samples were subjected to automated DNA extraction in a nucleic acid purifier (Concert, H8/HF16),the procedures of Automated Nucleic Acid Extractor are as follows:

1. Turn on the nucleic acid purifier and warm it up for 20 min before each experiment.
2. 2 Remove the T-rack and reagent strip rack from the nucleic acid purifier.
3. Place the reagent strips into the reagent strip rack of the nucleic acid purifier according to the diagram in the manual of the nucleic acid purifier.
4. Place the pre-treated sample tubes in lane 4 of the T-rack, the collection tubes in lane 1 of the T-rack, and the tips & tip sets in lane 2 of the T-rack.
5. Place the reagent strip holder into the Nucleic Acid Purifier and then into the T-rack so that the T-rack is pressed against the reagent strip.
6. Close the front door of the Nucleic Acid Purifier.
7. Click the "Start" button on the control panel of the Nucleic Acid Purifier to start the automatic calibration.
8. When the automatic calibration is completed, enter the code "102" of the reagent strip and click "Enter" to enter the next screen.
9. Press "1" (400 μL) to select the sample volume, and then click "Enter" to enter the next screen.
10. Confirm that the reagent strip code, sample type and sample volume are displayed correctly, and then click "Enter" to enter the next screen.
11. After confirming that the reagent strip rack and T-rack are correctly placed, click "Enter" to enter the next screen.
12. Press the numeric keys "1" (200 μL), "2" (150 μL), "3" (100 μL) or "4" (60 μL) to select the reagent strip. " (60 μL) to select the final elution volume and the instrument will start to run automatically.
13. After the instrument finishes running, the liquid in the collection tube is the extracted genomic DNA.

**Protocol of PCR amplification procedure**

1. Target fragment multiplex PCR reactions for samples.

(1).The specific primer multiplex PCR reaction system is as follows:

| **Components** | **Volume** |
| --- | --- |
| 10× buffer (TAKARA) | 1μL |
| dNTP (2.5mM) | 1.2μL |
| MgCl_2_ (25mM) | 0.6μL |
| Gene-specific primers (1μM) | 1μL |
| HotTaq 5U/μL (Takara) | 0.07μL |
| Bisulfite treatment of sample DNA | 1μL |
| ddH_2_O | 5.13μL |
| **Total volume** | **10μL** |

(2).Specific primer multiplex PCR reaction conditions:

|  | **Denaturation** | **Annealing** | **Extension** | **Holding** | **Cycle number** |
| --- | --- | --- | --- | --- | --- |
| 1 Step | 95°C 2min |  |  |  | 1× |
| 2 Step | 95°C 20s | 62°C 40s | 72°C 1min |  | 11× (-0.5°C/cycle) |
| 3 Step | 95°C 20s | 64°C 30s | 72°C 1min |  | 24× |
| 4 Step |  |  | 72°C 1min |  | 1× |
| 5 Step |  |  |  | 4°C | forever |

1. 1.5% agarose gel electrophoresis to confirm the validity of PCR amplification of the samples.

(4). Mixing of equal proportions of multiplex PCR products from each panel of the same sample.

(5). The mixed multiplex PCR products, diluted 10~20 times, are used as templates for the post-learning Index PCR step.

1. Addition of sample-specific tag sequences

Primers with Index sequences were used to introduce specific tag sequences compatible with the illumina platform to the end of the library by PCR amplification.

1. .The Index PCR reaction system is as follows:

| **Components** | **Volume（μL）** |
| --- | --- |
| 5× Reaction Buffer | 4μL |
| dNTP (2.5mM) | 2.4 μL |
| NGMPCRF (10μM)* | 0.8μL |
| NGMPCRR (4 μM)* | 2μL |
| Herculase® II Fusion DNA Polymerase | 0.2μL |
| PCR product after dilution | 2μL |
| ddH_2_O | 10.6μL |
| **Total volume** | 1. μL |

1. .Index PCR reaction conditions:

|  | **Denaturation** | **Annealing** | **Extension** | **Holding** | **Cycle number** |
| --- | --- | --- | --- | --- | --- |
| 1 Step | 95°C 2min |  |  |  | 1× |
| 2 Step | 95°C 20s | 60°C 40s | 72°C 1min |  | 12 |
| 3 Step |  |  | 72°C 2min |  | 1× |
| 4 Step |  |  |  | 4°C | forever |
